# Supplementary material for: Computational Screening of Newly Designed Compounds against Coxsackievirus A16 and Enterovirus A71
Source: Molecules. 2022 Mar 15;27(6):1908. doi: 10.3390/molecules27061908 (PMC8955072; doi:10.3390/molecules27061908)
Supplement: Supplementary file 1 [file molecules-27-01908-s001.zip › molecules-1561357-supplementary.pdf]

Supplementary Information

Computational Screening of Newly Designed Compounds Against Coxsackievirus A16 and Enterovirus A71

Amita Sripattaraphan[1], Kamonpan Sanachai[1], Warinthorn Chavasiri[2], Siwaporn Boonyasuppayakorn[3] Phornphimon Maitarad[4] and Thanyada Rungrotmongkol[1,5,*]

[1]*Structural and Computational Biology Research Unit, Department of Biochemistry, Faculty of Science, Chulalongkorn University, Bangkok, Thailand*
[2]*Department of Chemistry, Faculty of Science, Chulalongkorn University, Bangkok 10330, Thailand*
[3]*Applied Medical Virology Research Unit, Department of Microbiology, Faculty of Medicine, Chulalongkorn University, Bangkok 10330, Thailand*
[4]*Research Center of Nano Science and Technology, Shanghai University, Shanghai 200444, PR China*
[5]Ph.D. Program in Bioinformatics and Computational Biology, Faculty of Science, Chulalongkorn University, Bangkok 10330, Thailand

*Corresponding author: E-mail: thanyada.r@chula.ac.th, t.rungrotmongkol@gmail.com

Table of Content

1	Table S1. Chemical structures of rupintrivir and its 50 analogs	S3
2	Table S2 binding free energy based on the MM/PB(GB)SA method for rupintrivir and P2-m3 binding to EV-A71 and CV-A16 3Cpro	S10
3	Figure S1 Rupintrivir in the active site of EV-A71 3Cpro	S10
4	Figure S2 Superimposition of ligand at the binding site derived from the last 50 ns of run1 simulation	S11
5	Figure S3 The solubility of rupintrivir and P2-m3 from ADMET property	S12
6	Figure S4 Sequence alignment of EV-A71 and CV-A16 3C proteases	S12

1. Table S1 Chemical structures of rupintrivir and its 50 analogs

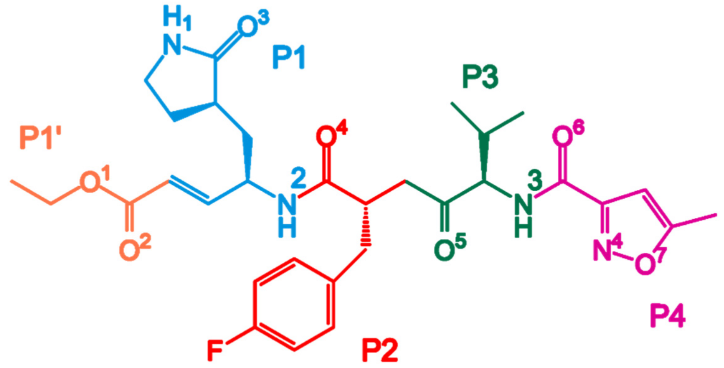

Code	P1’	P1	P2	P3	P4
rupintrivir	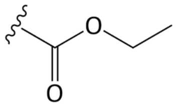	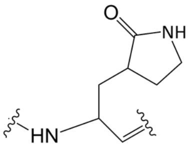	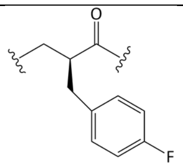	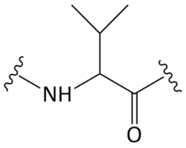	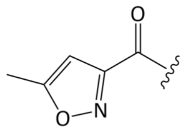
P1'-1	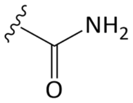				
P1'-2	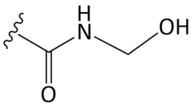				
P1-1		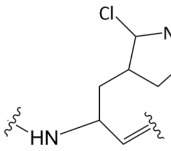			
P1-2		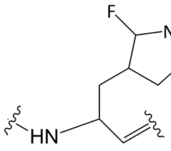			
P1-3		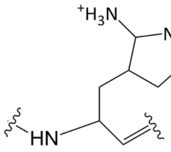			
P1-4		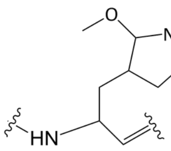			

Code	P1’	P1	P2	P3	P4
P1-5		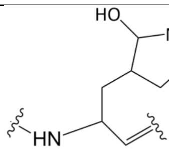			
P2-p1			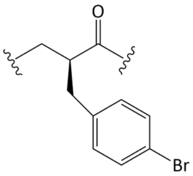		
P2-p2			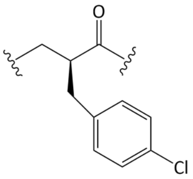		
P2-p3			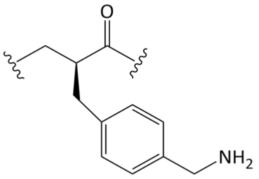		
P2-p4			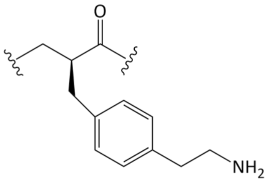		
P2-p5			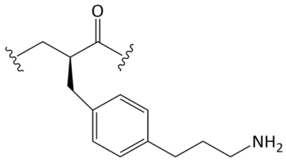		
P2-p6			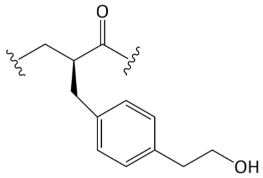		
P2-p7			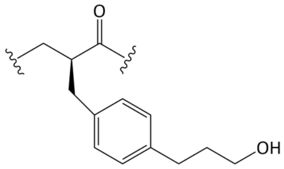		

Code	P1’	P1	P2	P3	P4
P2-p8			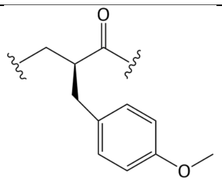		
P2-p9			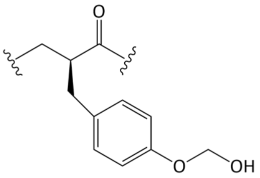		
P2-p10			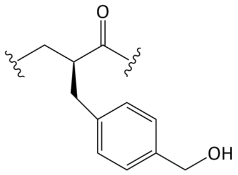		
P2-m1			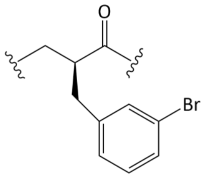		
P2-m2			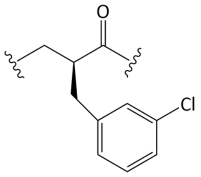		
P2-m3			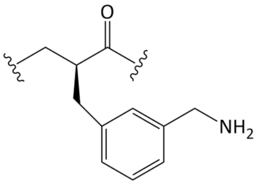		
P2-m4			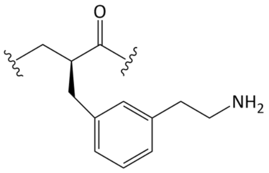		
P2-m5			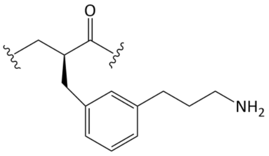		

Code	P1′	P1	P2	P3	P4
P2-m6			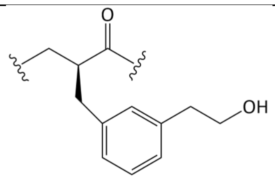		
P2-m7			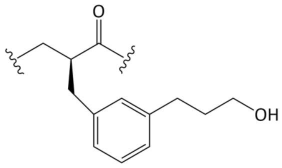		
P2-m8			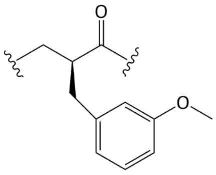		
P2-m9			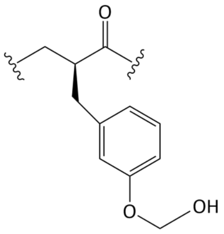		
P2-m10			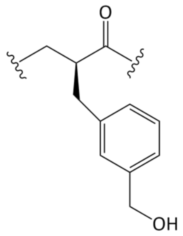		
P3-1				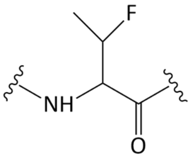	
P3-2				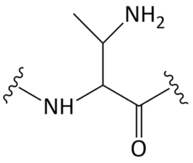	

Code	P1’	P1	P2	P3	P4
P3-3				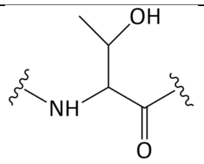	
P3-4				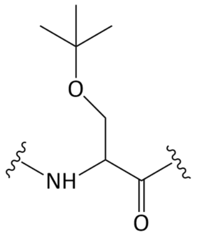	
P4-1					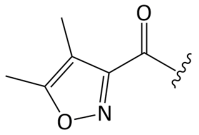
P4-2					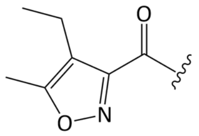
P4-3					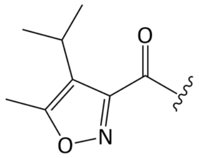
P4-4					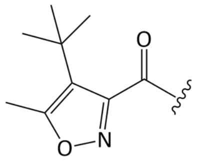
P4-5					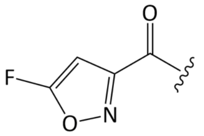
P4-6					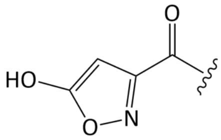

Code	P1′	P1	P2	P3	P4
P4-7					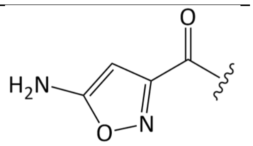
P4-8					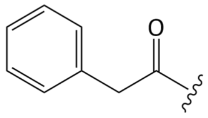
P4-9					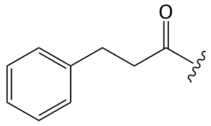
P4-10					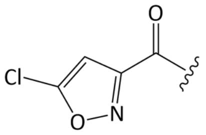
P4-11					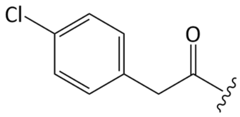
P4-12					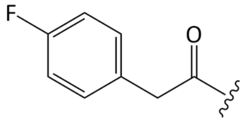
P4-13					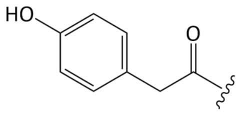
P4-14					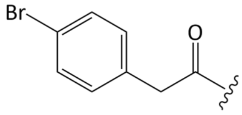
P4-15					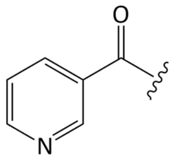
P4-16					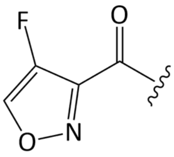

Code	P1’	P1	P2	P3	P4
P4-17					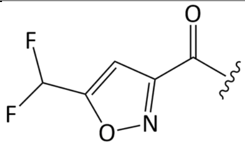
P4-18					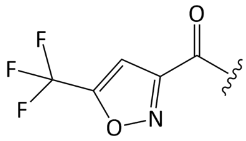
P4-19					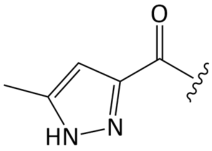

2. Table S2 Binding free energy based on the MM/PB(GB)SA method of rupintrivir and P2-m3

Compound		EV-A71		CV-A16	
		MM/PBSA	MM/GBSA	MM/PBSA	MM/GBSA
**Rupintrivir**	**Run1**	-10.1±1.5	-11.9±0.6	-7.8±0.5	-9.5±0.8
	**Run2**	-7.1±1.2	-9.1±1.0	-6.0±0.4	-8.5±0.9
	**Run3**	-6.4±1.0	-8.6±0.7	-8.9±0.9	-11.1±1.2
	**Mean±SD**	-7.9±2.0	-9.9±1.4	-7.6±1.1	-9.7±1.7
**P2-m3**	**Run1**	-17.2±0.6	-17.4±0.5	-11.8±0.3	-13.0±0.4
	**Run2**	-15.1±0.6	-15.6±0.3	-10.4±0.5	-12.7±0.3
	**Run3**	-15.0±0.8	-15.4±0.6	-10.1±0.7	-12.9±1.0
	**Mean±SD**	-15.8±1.2	-16.1±0.8	-10.8±0.9	-12.9±1.1

3. Figure S1 Rupintrivir in the active site of EV-A71 3Cpro

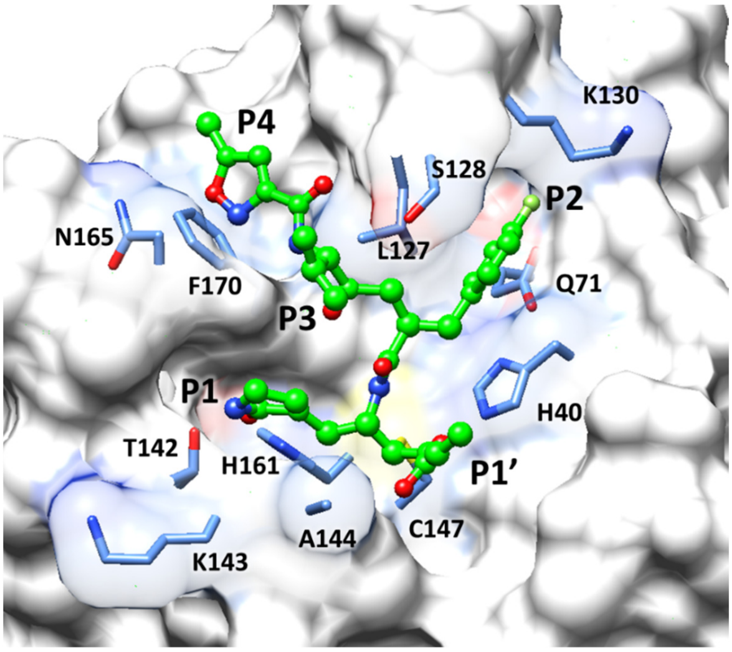

Figure S1 Rupintrivir (bond and stick model) binding at the active site of EV-A71 3Cpro, where its the surrounding residues (stick model) are shown and labeled

4. Figure S2 Superimposition of ligand at the binding site derived from the last 50 ns of run1 MD simulation

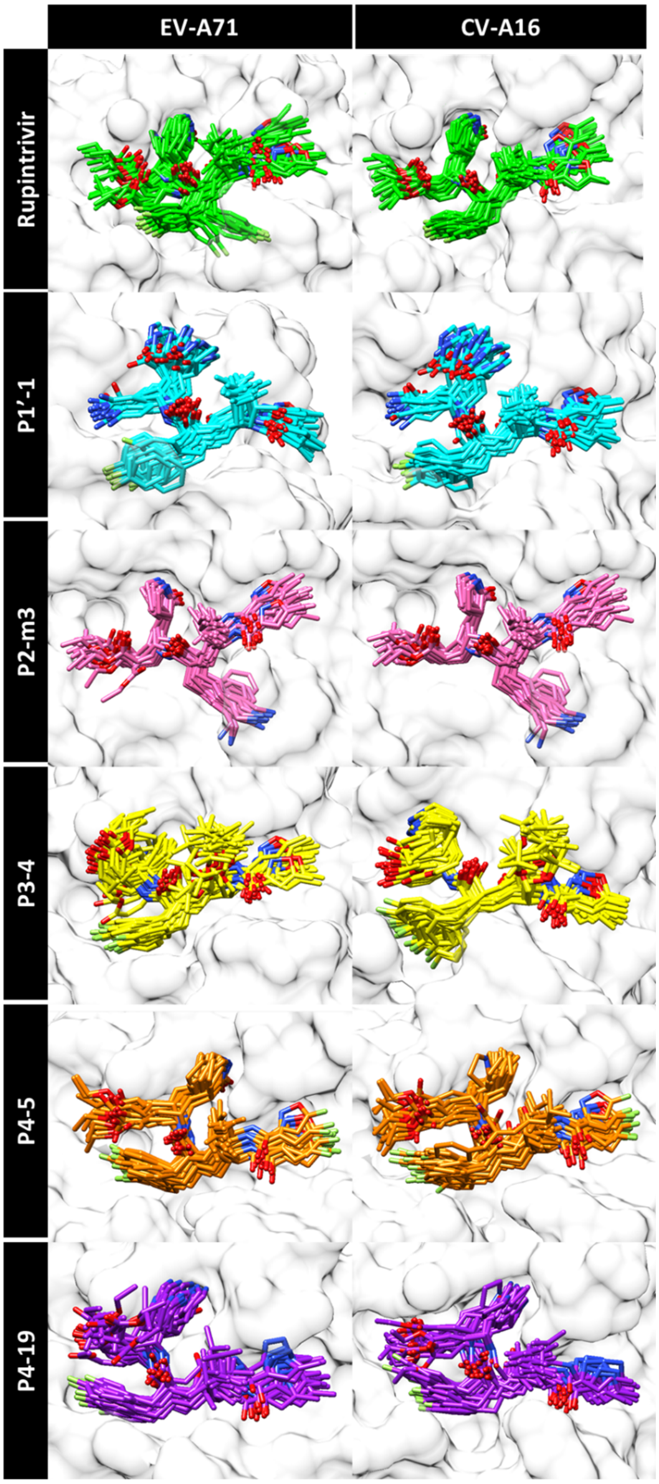

Figure S2 Superimposition of rupintrivir and 5 screened analogs at the binding pocket of EV-A71 and CV-A16 3Cpro derived from the last 50 ns of run1 MD simulation

5. **Figure S3**. The solubility of rupintrivir and P2-m3 from ADMET property

Compound	Solubility
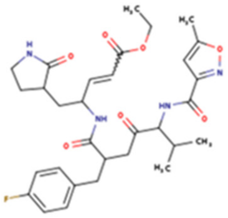 **Rupintrivir**	Log *S* (ESOL) -6.05<br>Solubility 5.36e-04 mg/ml ; 8.95e-07 mol/l<br>Class Poorly soluble
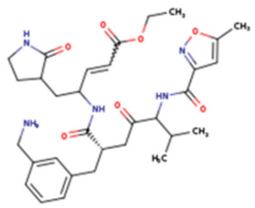 **P2-m3 analog**	Log *S* (ESOL) -3.72<br>Solubility 1.17e-01 mg/ml ; 1.92e-04 mol/l<br>Class Soluble

Figure S3. The solubility from ADMET property of rupintrivir and P2-m3. P2-m3 showed better solubility than rupintrivir

6. **Figure S4**. Sequence alignment of EV-A71 and CV-A16 3C proteases

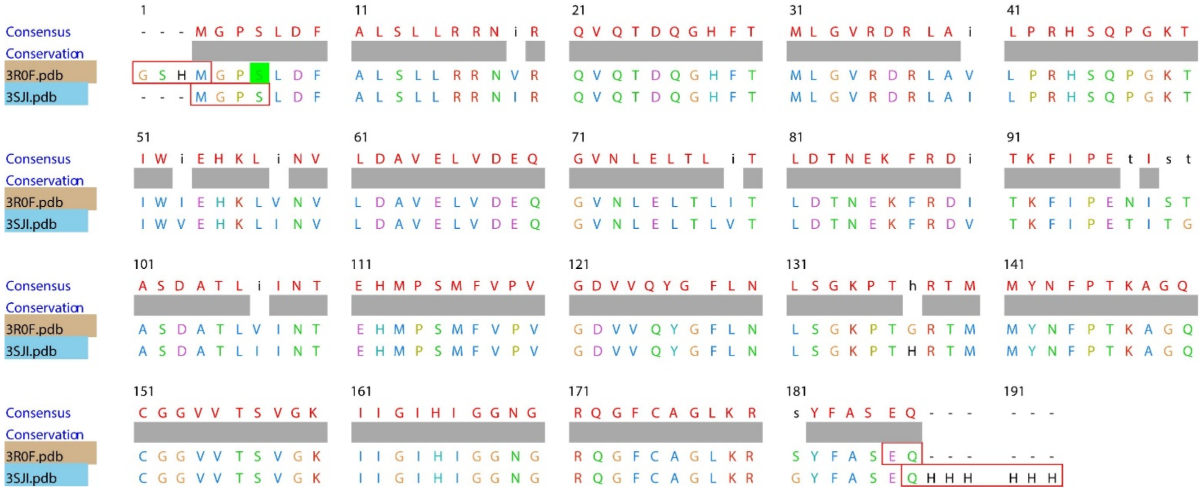

Figure S4. Sequence alignment of EV-A71 (PDB code: 3SJI) and CV-A16 (PDB code: 3R0F) proteases
